# Current Evidence for the Use of HIPEC and Cytoreductive Surgery in Gastric Cancer Metastatic to the Peritoneum

**DOI:** 10.3390/jcm12206527

**Published:** 2023-10-14

**Authors:** Anish J. Jain, Brian D. Badgwell

**Affiliations:** Department of Surgical Oncology, The University of Texas MD Anderson Cancer Center, Houston, TX 77030, USA

**Keywords:** HIPEC, cytoreductive surgery, gastric cancer, peritoneal carcinomatosis, laparoscopic HIPEC, intraperitoneal chemotherapy, peritoneal metastasis, metastatic gastric cancer

## Abstract

**Simple Summary:**

Advanced gastric cancer (GCa) is associated with poor prognosis due to the challenge of peritoneal disease, the most common site of metastasis at initial diagnosis, staging laparoscopy, and recurrence. Unfortunately, current standards of care for peritoneal metastasis (PM) in GCa are palliative therapies, despite hyperthermic intraperitoneal chemotherapy (HIPEC) being a proven standard of care for other peritoneal malignancies. Fortunately, results from studies investigating the impact of HIPEC in patients suffering from GCa with PM are encouraging. Additionally, several ongoing trials may offer further data and some centers have incorporated HIPEC into their treatment of patients with metastatic GCa. HIPEC can potentially confer improved survival outcomes in select patients with GCa and PM, who historically have dismal outcomes, and further well-designed clinical trials are warranted. This manuscript provides a comprehensive review of the current evidence supporting the use of HIPEC and cytoreductive surgery (CRS) in patients suffering from GCa with PM.

**Abstract:**

Gastric cancer (GCa) is an aggressive malignancy, representing the third leading cause of cancer mortality worldwide. The poor prognosis of GCa can be associated with the prevalence of peritoneal metastasis (PM). Current international and national GCa treatment guidelines only recommend palliative treatment options for patients with PM. Since the 1980s there have been multiple single arm trials, randomized controlled trials, and metanalysis investigating the use of cytoreductive surgery (CRS) and hyperthermic intraperitoneal chemotherapy (HIPEC) in patients with advanced GCa, with or without PM. Results from these studies have been encouraging, with some large-volume centers even incorporating HIPEC into their treatment algorithms for patients with advanced GCa. Additionally, there are several ongoing trials that, when completed, will increase our understanding of the efficacy of CRS & HIPEC in patients with GCa metastatic to the peritoneum. Herein we review the current evidence, ongoing trials, consensus guidelines, and future considerations regarding the use of CRS & HIPEC in patients suffering from GCa with PM.

## 1. Introduction

Gastric cancer (GCa) is the fifth most commonly diagnosed cancer in the world, but the third leading cause of cancer-related mortality worldwide [1,2,3]. In fact, only 25% of patients will survive 5 years [2,4]. The poor prognosis of GCa can be attributed to the challenges associated with peritoneal metastasis (PM).

The majority of patients with GCa are initially diagnosed with advanced disease, with PM present in 15–30% [3,5,6,7]. Alarmingly, even amongst patients that appear potentially resectable with less advanced disease on pre-therapy imaging, upwards of 30% are discovered to have PM at initial staging laparoscopy. Unfortunately, this rate has remained consistent over an 18-year time period (1995–2012), despite advances in imaging technologies [8]. Amongst patients with an initial negative staging laparoscopy who receive pre-operative therapy (chemotherapy or chemoradiation), approximately 10% will have PM at the time of the planned curative-intent resection [9]. Furthermore, of patients who ultimately undergo curative-intent resection, 40–60% will develop recurrence within the peritoneum [10]. The peritoneum therefore represents the most common site of metastatic disease at diagnosis, at pre-operative staging laparoscopy, and site of recurrence for patients with GCa [8,9,10,11].

Currently, the 2023 National Comprehensive Cancer Network (NCCN) guidelines for GCa recommend that patients with metastatic gastric cancer, including those with PM, receive only chemoradiation, systemic therapy, or the best supportive care. Venting gastrostomy and/or feeding tubes can be considered in these patients, but gastric resection should only be performed for palliation of refractory symptoms [12]. Additionally, several international guidelines recommend palliative intravenous chemotherapy as the only treatment for GCa with PM [13,14]. Regarding the use of hyperthermic intraperitoneal chemotherapy (HIPEC) in the treatment of GCa, the NCCN recommends HIPEC or laparoscopic HIPEC as a therapeutic alternative for carefully selected stage IV patients in the setting of ongoing clinical trials only [12]. An NCCN guideline update is currently in progress and recommendations regarding intraperitoneal therapy may have undergone modification by the publication of this review.

However, the use of HIPEC following cytoreductive surgery (CRS) has produced encouraging outcomes in the treatment of various other malignancies with PM [2]. HIPEC involves the continuous circulation of a heated, sterile chemotherapy continuing solution throughout the peritoneal cavity, often immediately after the removal of all macroscopic tumor deposits during CRS. Thus, it allows for the infusion of high-dose chemotherapy directly into the abdominal cavity, a site which systemic chemotherapy may not effectively treat due to the blood–peritoneal barrier [12]. Conversely, because of this blood–peritoneal barrier, high concentrations of chemotherapy can be administered directly into the peritoneum without penetration into the bloodstream, thus avoiding the toxic effects associated with the systemic administration of chemotherapy. In fact, the combination of CRS followed by HIPEC is the standard of care for several malignancies including peritoneal mesothelioma, ovarian cancer, and pseudomyxoma peritonei [15,16,17,18,19,20,21].

Whilst the survival rates of patients with GCa have improved, PM in patients with GCa is associated with poor survival rates of only months [3,22,23,24]. The dissemination of free tumor cells through blood or lymph in the abdominal cavity is considered one of the most common causes of PM in GCa, thus it is imperative that physicians find ways to eliminate free tumor cells in the abdominal cavity [25]. Unfortunately, only palliative treatment options are recommended for PM in GCa and the use of HIPEC has historically been controversial [12,13,14,26]. However, recent findings have increased enthusiasm in the use of CRS & HIPEC as a treatment option that can improve outcomes [12].

This review discusses the current evidence, ongoing trials, consensus guidelines, and future considerations regarding the use of CRS & HIPEC in patients suffering from GCa with PM. Pub med was utilized to review the existing literature from 2010 to 2023, only including clinical trials or prospective multi-institutional registry studies.

## 2. Rationale for HIPEC & CRS in Metastatic Gastric Cancer

Since the 1980s, there have been several randomized controlled trials (RCTs) and retrospective case–control studies investigating the use of HIPEC in patients with GCa [3]. A 2012 metanalysis based on 10 RCTs analyzed the outcomes for prophylactic HIPEC in patients with advanced GCa without distant metastasis who were randomized to undergo surgical resection alone versus surgical resection with HIPEC. While 7 RCTs used mitomycin C as the primary drug in HIPEC, the other 3 used 5-FU [27,28,29,30,31,32,33,34,35,36]. The metanalysis consisted of 1062 patients suffering from GCa with macroscopic serosal invasion, and it is important to note they did not have established peritoneal disease. In total, 544 underwent surgical resection alone, the control group, while the other 518 underwent resection with HIPEC. The metanalysis demonstrated a significant improvement in survival in patients who received HIPEC (RR 0.73 95% CI [0.64–0.83]; *p* < 0.001) compared to those who underwent resection alone. Additionally, the metanalysis reported that the use of HIPEC may potentially reduce the rate of peritoneal recurrence (RR 0.45 [0.28–0.72]; *p* = 0.001) compared to resection alone [25].

Similarly, a 2017 metanalysis evaluated 11 RCTs and 21 non-randomized control trials (NRCTs) that compared surgery and HIPEC to standard oncologic management in a total of 2520 patients with advanced-stage GCa, with or without PM [26,27,28,29,30,31,32,37,38,39,40,41,42,43,44,45,46,47,48,49,50,51,52,53,54,55]. For patients without PM (n = 1810), there was an improvement in 3- (RR 0.71 [0.53–0.96]; *p* = 0.03) and 5-year overall survival (OS) in the HIPEC group (RR 0.82 [0.7–0.96]; *p* = 0.01) compared to the control group. Additionally, patients without PM who underwent HIPEC had a reduced risk of overall disease recurrence (RR 0.73 [0.59–0.89]; *p* = 0.002) compared to their peers in the control group. In patients with PM, those who underwent HIPEC only had an improved 1-year survival (RR 0.67 [0.52–0.86]; *p* = 0.002) compared to the control group. Although, patients with PM who received HIPEC also had an increased median survival of 4.04 months compared to the control group (*p* < 0.01) [26].

Although these metanalyses consisted of investigations into the use of HIPEC for advanced GCa without PM, they offer encouraging insight into the role of HIPEC in improving survival, reducing recurrence, and controlling/addressing the peritoneal dissemination of advanced GCa [25,26]. Additionally, they provide some rationale for several notable clinical trials investigating the use of HIPEC in patients suffering from GCa with PM that warrant mentioning.

In 2011, Yang et al. published the results of a phase III RCT evaluating the efficacy and safety of CRS & HIPEC for the treatment of PM from GCa. A total of 68 patients with GCa and PM were randomized to either undergo CRS alone (n = 34) or CRS & HIPEC (n = 34), with a median follow-up of 32 months. Clinicopathologic characteristics including median age (51 vs. 50 years), median peritoneal carcinomatosis index (PCI) (15 vs. 15), and rates of PCI ≥ 20 (26.5% vs. 41.2%) were similar (all *p* > 0.05) in the CRS alone and CRS & HIPEC groups, respectively. Additionally, major perioperative characteristics were also similar (all *p* > 0.05) between the CRS alone versus the CRS & HIPEC group, including median operative time (4 h vs. 5 h), completeness of CRS score 0–1 (CC0–1) (58.8% vs. 58.8%), and rate of serious adverse events (11.7% vs. 14.7%). However, there was a significantly improved median OS in the HIPEC & CRS group (11.0 months, 95% CI [10.0–11.9]) compared to the CRS alone group (6.5 months [4.8–8.2]; *p* = 0.046). Additionally, on multivariate analysis, CRS & HIPEC was 2.6 times likely to improve survival (HR = 2.617, 95% CI [1.436–4.769]; *p* = 0.002) compared to CRS alone. The investigators concluded that CRS & HIPEC may improve survival in patients suffering from GCa with synchronous PM [41].

In 2014, Rudloff et al. published the results of the GYMSSA trial, a prospective RCT investigating the impact of systemic chemotherapy versus multimodality therapy (CRS, HIPEC, and systemic chemotherapy) on OS in patients with gastric carcinomatosis. Ultimately 17 patients were enrolled, with 9 randomized to the GYMS arm (systemic chemotherapy, HIPEC, and CRS) and the other 8 to the SA arm (systemic chemotherapy only). The median OS in the SA arm was 4.3 months with no patient surviving beyond 11 months, but the median OS in the GYMS arm was 11.3 months with four patients surviving beyond 12 months. Furthermore, all patients in the GYMS arm who survived beyond 12 months achieved complete cytoreduction and had an initial PCI ≤ 15. Although the study had to be closed prematurely due to slow accrual these findings are noteworthy, suggesting that maximal CRS & HIPEC combined with systemic chemotherapy can result in prolonged survival in carefully in selected patients with gastric carcinomatosis and limited disease burden [40].

The CYTO-CHIP study analyzed 277 patients with PM from GCa treated with CRS with or without HIPEC at 19 French centers between 1989–2014. The study also grouped patients into those with poorly cohesive carcinoma (PCC, n = 188), who had worse OS regardless of treatment, and those with non-PCC (n = 89). Multivariate analysis showed that in all patients, regardless of histology (PCC vs. non-PCC), undergoing CRS & HIPEC versus CRS alone was associated with improved OS (HR = 0.52 [0.38–0.71], *p* < 0.001). In the PCC group specifically, the OS rates at 1, 3, and 5 years were 64.9%, 14.2%, and 7.1% percent in those who underwent CRS & HIPEC versus 45.3%, 9.4%, and 1.9% in those who underwent CRS alone (*p* = 0.024) In the non-PCC group, OS rates at 1, 3, and 5 years were 76.3%, 48.3%, and 38.6%, respectively, for those who underwent CRS & HIPEC versus 54.5%, 22.0%, and 18.4% in those who underwent CRS alone (*p* = 0.008). Additionally, for the entire study population, the recurrence-free survival (RFS) rates at 1, 3, and 5 years were 46.3%, 14.9%, and 10.5%, respectively, in the CRS-HIPEC group versus 35.1%, 6.1%, and 3.6% in the CRS alone group (*p* =0.009). Furthermore, in patients who underwent CRS & HIPEC, OS was improved with low (0–6) and intermediate (7–12) PCI levels (*p* = 0.018). Thus, the investigators concluded that CRS & HIPEC offer acceptable outcomes for select patients with gastric PCC and long-term survival for those with non-PCC GCa [56].

Between 2014 and 2016, Badgwell et al. conducted a single-arm phase II study investigating the use of neoadjuvant laparoscopic HIPEC in patients with GCa and positive peritoneal cytology or PM. Evaluation for trial eligibility was performed after completion of systemic chemotherapy, with inclusion limited to patients with low volume PM and no solid organ metastasis since CRS was not a part of this trial. Patients underwent laparoscopic HIPEC at least 3 weeks after completing systemic chemotherapy, and it could be performed up to five times with a minimum of 3 weeks between procedures. Patients with negative peritoneal washings, no laparoscopic evidence of carcinomatosis, and no imaging evidence of solid organ metastases after neoadjuvant laparoscopic HIPEC procedures would be offered gastrectomy. Nineteen patients were enrolled and treated with a total of 38 laparoscopic HIPEC procedures. The median follow-up was 18.9 months, with a median OS of 30.2 months from the date of diagnosis of metastatic disease and 20.3 months from the first laparoscopic HIPEC. Seven patients (37%) had negative peritoneal cytology and no evidence of peritoneal carcinomatosis after their final HIPEC, with five (26%) electing to proceed with gastrectomy. The median OS for the five patients who underwent gastric resection was 29 months. Although the small cohort size of the trial precluded statistical analysis for the variables associated with progressing to resection, an encouraging number of patients could be offered gastrectomy and the results warranted future studies [57].

In 2020, Blum et al. published the results of the first phase I trial of triplet-drug (cisplatin, mitomycin, and paclitaxel) HIPEC regimen as a treatment for patients suffering from GCa with positive cytology or carcinomatosis. Between 2017 and 2018, twenty-seven patients underwent triplet-drug laparoscopic HIPEC, with no CRS or repeat laparoscopic HIPEC performed. Although the primary objective of the study was to determine safety and the maximum tolerated dose, promising survival rates were recorded as the secondary outcome. The 1- and 2-year OS rates from the date of metastatic disease were 73.9% and 58.1%, respectively. Additionally, while electrolyte abnormalities were common, systemic toxicity was uncommon. Once again, these results warrant further research into the use of HIPEC for advanced gastric cancer [58].

In 2021, findings from the GASTRIPEC trial based in Germany, another multicenter RCT investigating the impact of CRS & HIPEC in patients suffering from GCa with PM, were presented at the European Society of Medical Oncology Congress. Between 2014 and 2018, 105 patients were randomized to either undergo curative-intent CRS alone (CRS-A arm, n = 53) or CRS with HIPEC (CRS + H arm, n = 52), with all patients receiving pre- and postoperative systemic chemotherapy. Ultimately 55 patients stopped treatment prior to surgery due to either disease progression or death, and the median OS was 14.9 months in both arms. These findings highlight the limited efficacy of systemic therapy in the treatment of PM in GCa. However, the progression-free survival was significantly improved in the CRS + H arm (7.1 months) compared to the CRS-A arm (3.5 months; *p* = 0.047). Additionally, the distant metastasis-free survival was also improved in the CRS + H arm (10.2 months) compared to the CRS-A arm (9.2 months). Although the OS was similar in both arms, the investigators concluded that further investigations into the use of HIPEC in metastatic GCa were warranted given the improved progression and distant metastasis-free survival [59].

Between 2016 and 2019, Badgwell et al. conducted a single-arm phase II trial investigating the use of laparoscopic HIPEC followed by cytoreduction, gastrectomy, and HIPEC in patients with GCa and positive cytology or carcinomatosis. Patients were required to complete systemic chemotherapy and have undergone diagnostic laparoscopy with laparoscopic HIPEC prior to enrollment. At least 4 weeks after enrollment, patients would undergo surgical resection consisting of gastrectomy, CRS, and HIPEC followed by surveillance. Twenty patients were treated in the trial with a median PCI of 2 prior to surgical resection and a median follow up of 33.5 months. The median OS from the date of diagnosis of metastatic disease was 22.1 months; median OS from the date of first laparoscopic HIPEC was 17.4 months; and median OS from the date of cytoreduction, gastrectomy, and HIPEC was 16.1 months [60]. Similar to older trials and metanalyses, the results of this most recent trial have continued to provide further evidence rationalizing continued study and utility of CRS & HIPEC in patients with advanced GCa [25,26,40,41,56,57,58,59,60].

## 3. Ongoing Clinical Trials

There are still several notable ongoing trials investigating the utility of HIPEC as a treatment for patients with advanced-stage GCa. Results from these trials will offer perhaps the most definitive and impactful data on the impact of HIPEC in patients suffering from GCa with PM.

The GASTRICHIP trial is a prospective phase III RCT that commenced in 2014 with the purpose of evaluating the effects of HIPEC with oxaliplatin on patients with GCa involving the serosa and/or lymph nodes and/or with positive cytology at peritoneal washing. Patients with distant solid organ metastasis (liver, lung, ovaries, etc.), tumoral infiltration of the head or body of the pancreas, the existence of macroscopic peritoneal implants, or clinical significant ascites (>500 mL), will be excluded. Included patients will either be randomized to curative gastrectomy with D1-D2 lymph node dissection and HIPEC with oxaliplatin (Arm A) or to curative gastrectomy with D1-D2 lymph node dissection only (Arm B). The primary endpoint will be overall survival from the date of surgery to the date of death or to the end of follow-up (5 years). Secondary endpoints include 3- and 5-year RFS, recurrence sites, morbidity, and quality of life. The study has been powered to include 306 participants, which would make it the largest and potentially most impactful study addressing HIPEC in Western populations suffering from advanced GCa [60,61].

The PERISCOPE I trial demonstrated the safety and feasibility of CRS & HIPEC with oxaliplatin and normothermic docetaxel in patients with GCa and limited PM or positive cytology [62]. However, the ongoing PERISCOPE II trial aims to evaluate the efficacy of the aforementioned treatment. It is a multicenter, double-armed phase III RCT that will compare the OS between patients with GCa and limited PCa and/or positive peritoneal cytology treated with palliative systemic chemotherapy (standard treatment arm) versus gastrectomy, CRS, and HIPEC (experimental treatment arm) after 3–4 cycles of neoadjuvant chemotherapy. Patients will be included if their primary GCa tumor is considered resectable, there is no disease progression during neoadjuvant chemotherapy, distant metastases are absent, and the PCI < 7 [63]. By comparing palliative chemotherapy to gastrectomy with CRS & HIPEC, the PERISCOPE II trial will directly address the most prominent question in the ongoing debate surrounding the utility of CRS & HIPEC in GCa with PM: is CRS & HIPEC a superior treatment to the current standard of care for patients suffering from advanced GCa with PM [60]?

## 4. Current Incorporation of HIPEC for PM in GCa

The results of the ongoing PERISCOPE II and GASTRICHIP trials will have a significant impact on the use of HIPEC for advanced GCa, and potentially alter current treatment recommendations for patients with PM. However, there have been efforts to incorporate CRS & HIPEC into existing treatment algorithms for patients suffering from GCa with PM.

In 2018, The Chicago Consensus Working Group (CCWG) developed multidisciplinary recommendations for the management of GCa with PM based on the best available evidence at the time. For synchronous PM from GCa, the CCWG recommended systemic chemotherapy initially followed by restaging imaging and the consideration of laparoscopy. Surgical intervention could then be considered in patients with stable disease or improvement, no extraperitoneal disease, no distant nodal disease, and good functional status. Surgical intervention would consist of gastrectomy with or without CRS and intraperitoneal chemotherapy, based on the PCI and response to systemic chemotherapy. In patients with GCa who developed metachronous PM, the CCWG recommended systemic chemotherapy followed by restaging in patients with a low metastatic burden or a long disease-free interval after the administration of systemic therapy. In patients with stable or responsive disease on restaging, surgical intervention could be considered. Regarding the use of CRS & HIPEC in this patient population, the CCWG recognized the need for multi-institutional and randomized trials within the United States. They recommended that, while awaiting results of the previously mentioned RCTs outside the US, patients should be considered for current non-randomized trials and currently available prospective registries [64]. These guidelines are also currently under revision and modification to provide an updated summary of treatment options.

**Figure 1:**
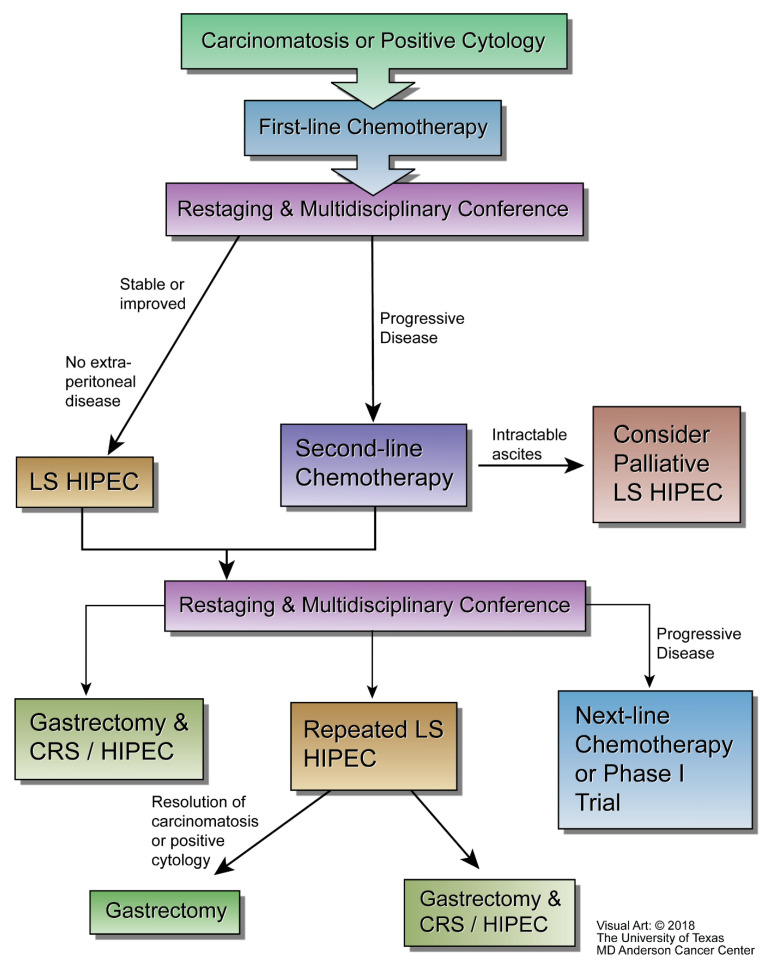
MD Anderson’s integration of laparoscopic HIPEC into the treatment algorithm for patients with gastric adenocarcinoma metastatic to the peritoneum (courtesy of the University of Texas MD Anderson Cancer Center).

At The University of Texas MD Anderson Cancer Center (MD Anderson), the multidisciplinary GCa group considers laparoscopic HIPEC for patients with a histological diagnosis of gastroesophageal or gastric cancer with low volume PM and/or positive peritoneal cytology, no other sites of metastasis, an ECOG performance status ≤ 2, adequate renal function (serum creatinine ≤ 1.5 mg/dL), adequate hematological function (leukocytes > 2000/µL, neutrophils > 1200/µL, platelets > 100,000/µL), and adequate hepatic function (liver function enzymes within 5 times the institutional upper limit of normal). All patients are treated with first-line chemotherapy prior to consideration for laparoscopic HIPEC in a multidisciplinary GCa conference and restaging. Patients with stable or improved disease would be eligible to undergo laparoscopic HIPEC at a minimum of 3 weeks after the last dose of systemic chemotherapy. After laparoscopic HIPEC patients would be re-staged and discussed at multidisciplinary conference again and either undergo surgical resection (gastrectomy, CRS, and HIPEC), repeat laparoscopic HIPEC, or next-line chemotherapy, or a phase I clinical trial. MD Anderson’s multidisciplinary treatment algorithm for patients suffering from GCa with PM is shown in Figure 1 below [65].

In 2019, Newhook et al. retrospectively reviewed the records of all patients with a history of gastric or gastroesophageal junction adenocarcinoma metastasis limited to the peritoneum treated with laparoscopic HIPEC at MD Anderson from June 2014 to January 2017. The analysis included 44 patients, including 19 from a previous phase II trial (NCT02092298), who underwent a total of 71 laparoscopic HIPEC procedures [60,65]. The median number of laparoscopic HIPEC procedures performed per patient was one (range 1–5 procedures). Three patients (7%) underwent palliative laparoscopic HIPEC for intractable ascites. Eleven patients (25%) ultimately underwent gastrectomy, with the majority (n = 10, 91%) doing so after negative cytology and resolution of PM. Thus, these patients underwent a curative-intent resection to prolong survival. This retrospective review illustrated the repeatability and feasibility of neoadjuvant laparoscopic HIPEC as part of a standardized treatment algorithm for patients with advanced GCa [65].

## 5. Future Directions

Current data from various metanalyses, single-arm trials, and RCTs, bolster the rationale for the use of CRS & HIPEC to improve the historically poor outcomes in patients suffering from GCa metastatic to the peritoneum. Results from ongoing trials will offer impactful data that will further enlighten physicians about the utility of CRS & HIPEC in these patients, especially in Western populations [60,61,63]. However, many questions must still be addressed including optimal timing, regimen, dosing, and eligibility criteria for CRS & HIPEC [4]. Perhaps the most important question that must be answered before CRS & HIPEC can be accepted as an alternative is how efficacious it is compared to current standard of care palliative chemotherapy? The highly anticipated PERISCOPE II trial, which is powered for 226 participants and has currently accrued at least 62, should provide insight into this [66]. Future RCTs also comparing the standard of care to CRS & HIPEC can legitimize it as a viable treatment option for GCa with PM, which could lead to future RCTs comparing HIPEC regimens thus addressing many of the aforementioned questions.

Other novel techniques are also being developed to deliver intraperitoneal chemotherapy to patients ineligible for and/or unable to withstand HIPEC. This includes pressurized intraperitoneal aerosol chemotherapy (PIPAC), which uses aerosolized chemotherapy to produce an increased tumor drug concentration with increased tissue penetration. In PIPAC, laparoscopic access is obtained, the abdomen insufflated to 12 mmHg pneumoperitoneum, and a nebulizer is introduced into the abdomen via a trocar. The nebulizer can deliver aerosolized, intraperitoneal chemotherapy creating therapeutic capnoperitoneum [67]. Animal models have demonstrated that the increased intrabdominal pressure can improve the concentration, penetration, and antitumor effects of intraperitoneal chemotherapy compared to conventional intraperitoneal or intravenous chemotherapy [68].

While advances in intraperitoneal chemotherapeutic techniques can offer hope, it is important to remember the words of the late Dr. Blake Cady, “Biology is King. Selection is Queen. Technical Maneuvers are the Prince and Princess” [69]. The success of CRS, HIPEC, and even PIPAC, in the treatment of metastatic GCa can be enhanced by an increased understanding of individual GCa tumor biology. The genetic profiling of tumors via large-scale next-generation sequencing is an emerging field that may allow physicians to administer optimal drug regimens based on individual patients’ tumor biologies [70]. For example, Ajani et al. have recently demonstrated that the yes-associated protein 1 (YAP1) oncogene is highly upregulated in malignant PM cells in GCa and appears to be a metastatic driver. Patient-derived PM cells, patient-derived xenograft (PDX), and patient-derived orthotopic (PDO) models were used to study the function of YAP1 in vitro and in vivo. The inhibition of YAP1 suppressed tumor growth in PDX models and blocked PM in PDO models, providing a strong rationale to target it in clinical settings [71]. Additionally, the ongoing PERICLES clinical trial aims to create a personalized circulating-tumor-DNA (ctDNA) test to guide treatment for patients with gastrointestinal cancer with peritoneal carcinomatosis. The establishment of a successful cell-free ctDNA test could aid in disease prognostication, assessment of response to therapy, and the identification of optimal treatments [4,72,73].

## 6. Conclusions

GCa metastatic to the peritoneum is a clinical predicament associated with extremely poor prognosis and despite its prevalence, current international treatment guidelines only recommend palliative chemotherapy. CRS & HIPEC, the standard of care for PM from other malignancies is only recommended in the setting of clinical trials for PM from GCa. However, since the 1980s, multiple RCTs, single-arm trials, and metanalyses worldwide have demonstrated the impact of CRS & HIPEC in the treatment of advanced GCa. Ongoing trials will offer further insight into the efficacy of CRS & HIPEC for GCa metastatic to the peritoneum. Additionally, at MD Anderson neoadjuvant laparoscopic HIPEC has been incorporated into a multidisciplinary treatment algorithm for GCa with PM, with several patients ultimately undergoing curative intent resections.

Additional RCTs comparing the efficacy of CRS & HIPEC to palliative chemotherapy may result in its incorporation into standard treatment guidelines. If CRS & HIPEC become incorporated into treatment guidelines, additional RCTs are needed to determine the most effective regimen, timing, and patient selection criteria. The current evidence presented in this review provides a strong rationale for the use and further study of CRS & HIPEC as a treatment option for patients suffering from GCa metastatic to the peritoneum.
